# Supplementary material for: Evidence for fungi and gold redox interaction under Earth surface conditions
Source: Nat Commun. 2019 May 23;10:2290. doi: 10.1038/s41467-019-10006-5 (PMC6533363; doi:10.1038/s41467-019-10006-5)
Supplement: Supplementary file 1 — Supplementary Information [file 41467_2019_10006_MOESM1_ESM.pdf]

Supplementary information

Evidence for fungi and gold redox interaction under Earth surface conditions

Bohu *et al*.

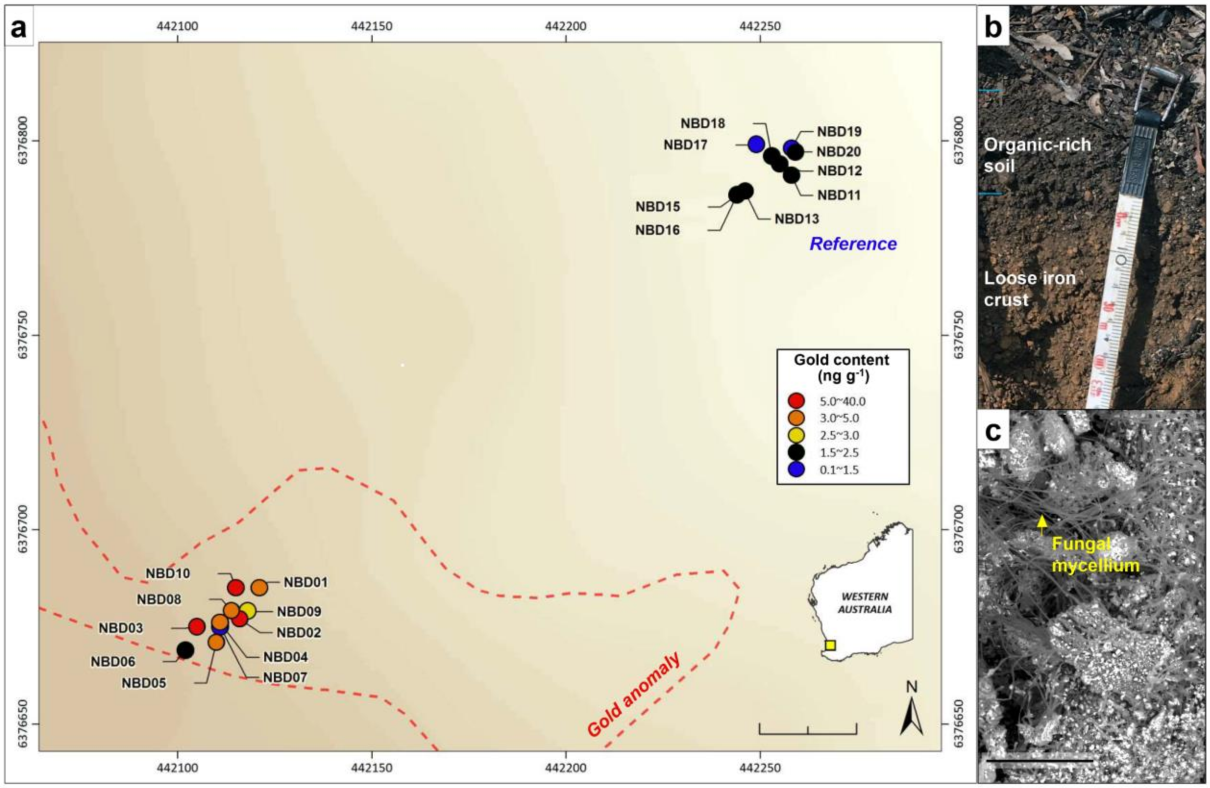

Supplementary Figure 1. The geological setting of the secondary gold deposit in the Golden Triangle Gold Prospect, Boddington, Western Australia. (a) Schematic map of sampling sites. The boundary of the buried secondary gold deposit is indicated by a dashed line. Dots represent sampling sites in the gold anomaly and the reference area. Each dot is labeled with a sample ID. The concentration of gold at each sampling site is depicted by a color gradient. Sampling sites NBD02, NBD03, and NBD10 with *in situ* gold concentrations 1.5-fold greater than or equal to the median (3.54 ng g^{-1}) were determined as gold hotspots. Scale bar, 25 m. (b) Profile of the surface regolith in the Golden Triangle Gold Prospect. The dark brown layer is organic-rich soil, and the lighter brown layer is loose iron crust. (c) Scanning electron micrograph of abundant fungal mycelia (yellow arrow) in the organic-rich soil. Scale bar, 300 μm.

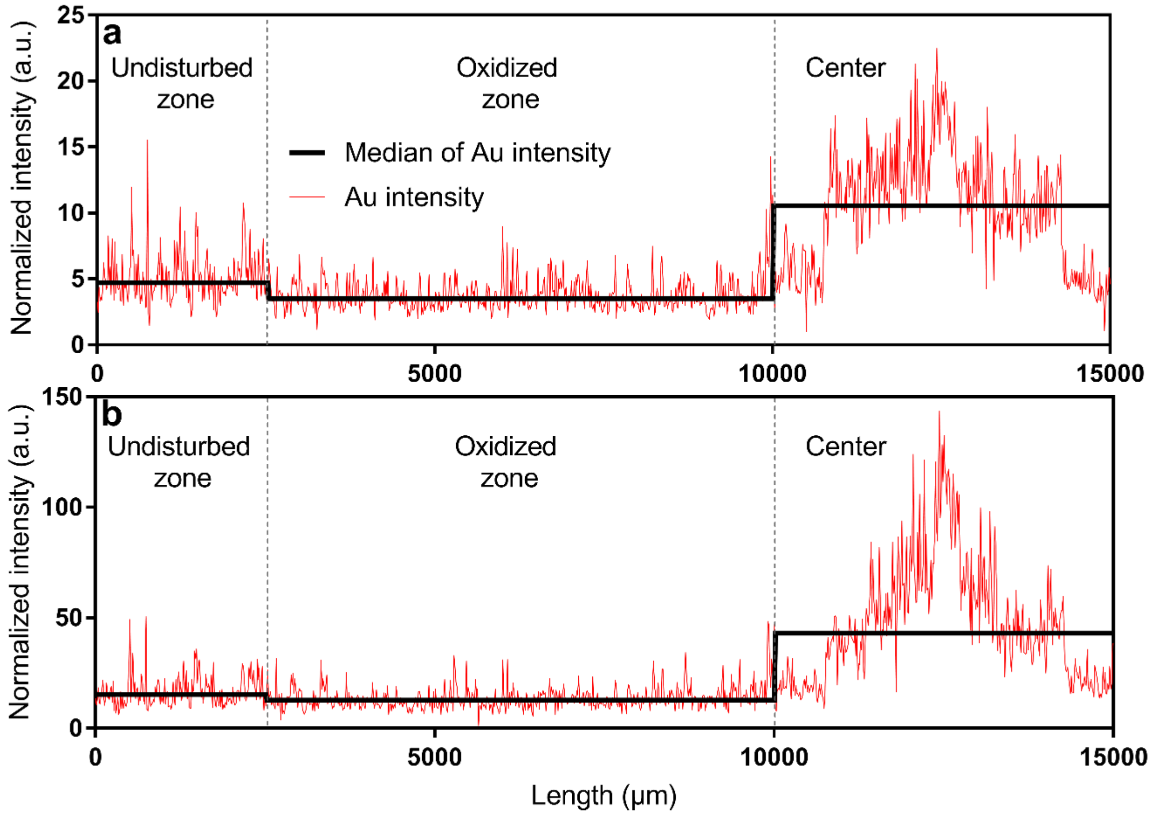

Supplementary Figure 2. Gold distributions in PYG agar plate, determined using LA-ICP-MS. The intensities were normalized to ^{39}K (a) and ^{25}Mg (b), respectively.

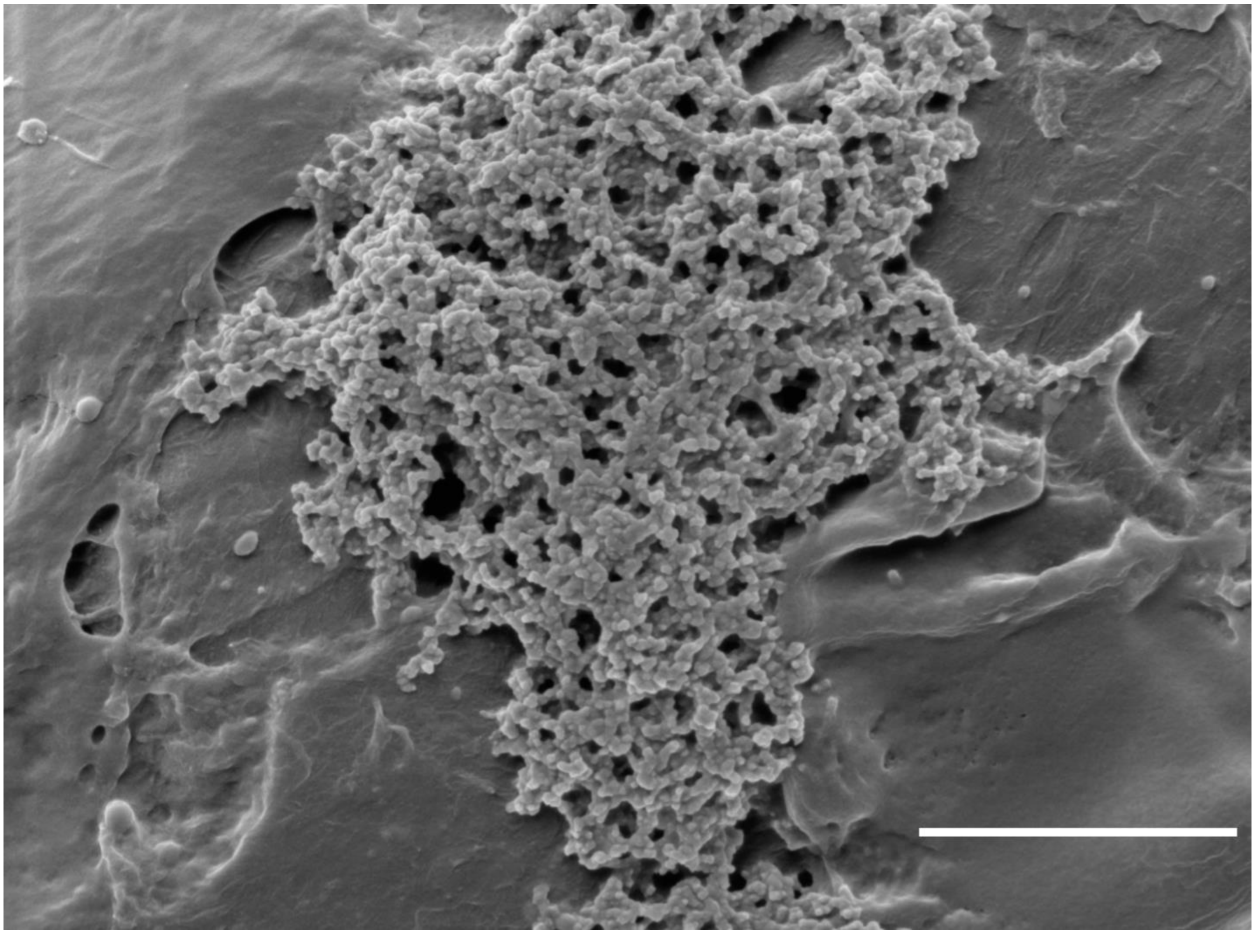

Supplementary Figure 3. SEM of gold complexes on the surface of a sterilized cotton fiber. Scale bar, 2.5µm.

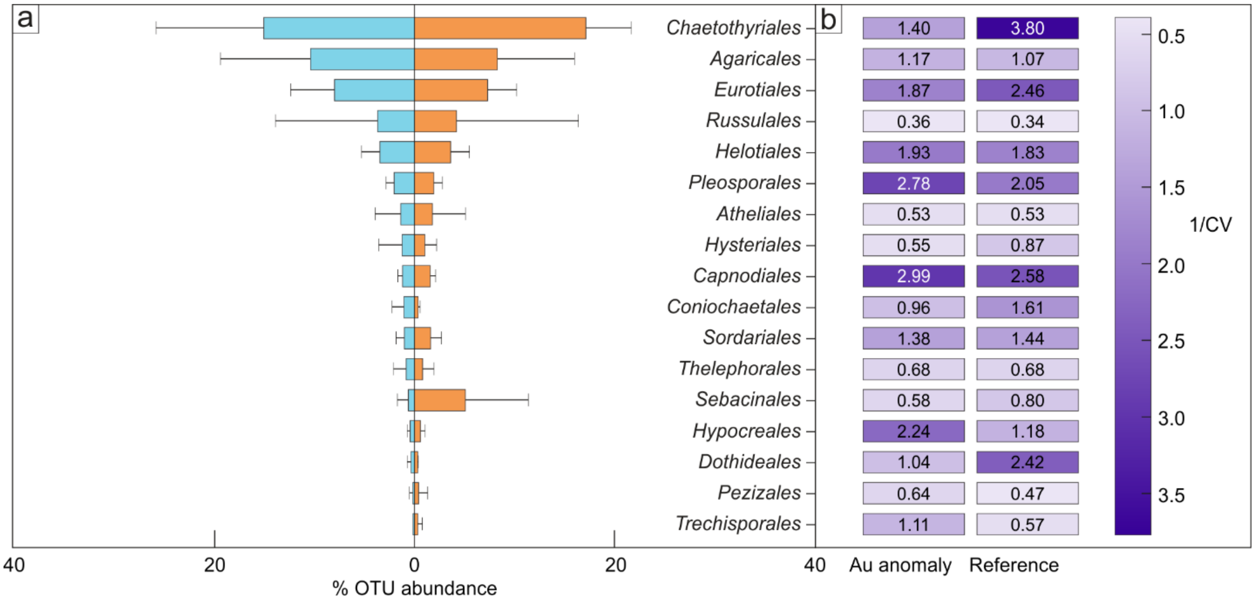

Supplementary Figure 4. Relative abundance and variability of each fungal OTU in the gold anomaly and the reference area. (a) Fungal abundance (order level) in the gold anomaly (blue bar) and reference area (orange bar). Fungal community compositions (OTU assignment: distance cut-off 0.03) were retrieved from the Illumina MiSeq sequencing of fungal rRNA gene ITSs. The horizontal axis represents the sequence percentage of each OTU. Error bars represent standard deviation. (b) Coefficients of variation (CV) of fungal abundance were calculated based on the mean OTU abundance and standard deviation. The gradient bar is the inverse of the CV (1/CV).

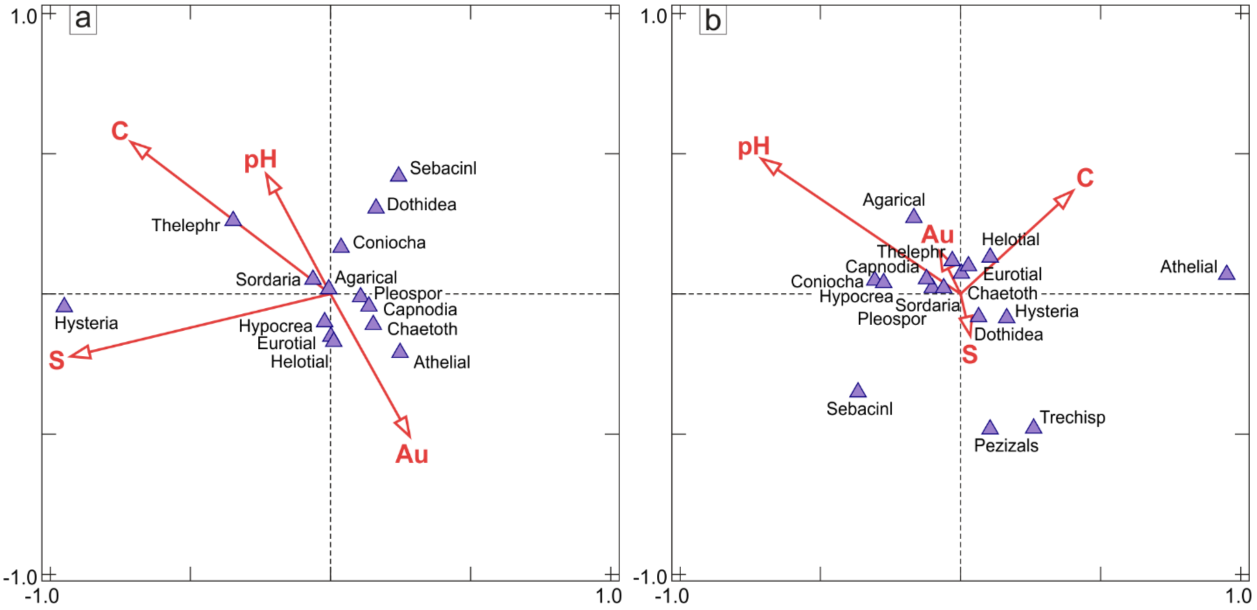

Supplementary Figure 5. Relationships between environmental variables and fungal community structure, analyzed using CCA with Monte Carlo permutation tests. Fungal community structure (OTU assignment: distance cut-off 0.03) was retrieved from Illumina MiSeq sequencing of fungal rRNA gene ITSs. (a, b) Distribution of fungal communities in the gold anomaly (a) and reference area (b). Explanatory variables accounted for 63.9% (a) or 39.2% (b) of sample variation. The eigenvalues of the first and second axes in panel (a) were 0.017 and 0.0075. In panel (b), the eigenvalues of the first and second axes were 0.0092 and 0.0045. Names of all microbial groups (taxa) were truncated to the first eight characters due to software limitations.

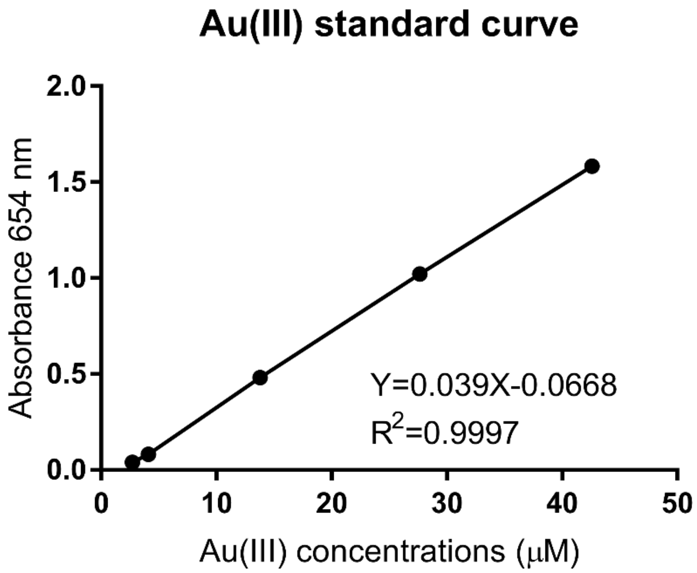

Supplementary Figure 6. The standard curve for Au(III) colorimetric measurement.

Supplementary Table 1. The *in situ* gold concentrations and experiment design of the samples

Sample ID	Coordinates (UTM WGS84)		Gold concentrations (ng/g)	Geochemical analysis[a]	Microcosm	Gold-oxidizing fungi isolation	MiSeq sequencing (fungal rRNA ITS)	MiSeq sequencing (bacterial 16S rRNA gene)
Gold anomaly								
NBD01	Zone 50 H	442121mE 6376685mN	3.84	●			●	●
NBD02	Zone 50 H	442116mE 6376677mN	40.4	●			●	●
NBD03	Zone 50 H	442105mE 6376675mN	9.91	●	●	●	●	●
NBD04	Zone 50 H	442111mE 6376676mN	3.15	●			●	●
NBD05	Zone 50 H	442110mE 6376671mN	4.70	●			●	●
NBD06	Zone 50 H	442102mE 6376671mN	1.89	●			●	
NBD07	Zone 50 H	442111mE 6376675mN	0.56	●			●	●
NBD08	Zone 50 H	442114mE 6376679mN	3.24	●	●	●	●	●
NBD09	Zone 50 H	442118mE 6376679mN	2.52	●			●	●
NBD10	Zone 50 H	442115mE 6376685mN	6.33	●	●	●	●	●
Reference								
NBD11	Zone 50 H	442258mE 6376791mN	2.22	●			●	●
NBD12	Zone 50 H	442255mE 6376794mN	1.59	●	●		●	●
NBD13	Zone 50 H	442246mE 6376787mN	2.20	●			●	●
NBD15	Zone 50 H	442244mE 6376786mN	1.33	●			●	
NBD16	Zone 50 H	442245mE 6376786mN	2.31	●			●	●
NBD17	Zone 50 H	442249mE 6376799mN	0.51	●	●		●	●
NBD18	Zone 50 H	442253mE 6376798mN	1.73	●	●		●	●
NBD19	Zone 50 H	442258mE 6376798mN	1.13	●			●	
NBD20	Zone 50 H	442259mE 6376797mN	2.20	●			●	

a, a black dot means the sample was applied in the experiment.

Supplementary Table 2. Linear regression analysis of hyphal extension

	**Sucrose**		**Lignin**	
	No gold	With gold	No gold	With gold
**Best-fit values ± SE**				
Slope	0.070±0.005	0.078±0.0005	0.081±0.001	0.088±0.0005
Y-intercept	-12.38±1.9	-2.05±0.2	-5.45±0.5	-6.73±0.2
X-intercept	177.90	26.36	66.96	76.67
**Goodness of Fit**				
R square	0.9947	0.9999	0.9990	0.9999
**Equation**				
	Y = 0.07×X - 12.4	Y = 0.078×X - 2.1	Y = 0.081×X - 5.5	Y = 0.088×X - 6.7
**Differences of the linear extension rates**				
P value	0.10		0.01	
Significance (*P* < 0.01)	NO		NO	
**Lag-phase (h)**				
	0-195.7	0-38.6	0-83.0	0-79.9

Supplementary Table 3. Sequence frequency of the fungal OTUs in order-level

OTUs (order-level)	Gold anomaly				Reference area			
	Mean	SD	CV	Relative abundance	Mean	SD	CV	Relative abundance
Trechisporales	0.0506	0.0455	0.8992	1.1121	0.3179	0.5586	1.7572	0.5691
Pezizales	0.1659	0.2578	1.5539	0.6435	0.4432	0.9385	2.1176	0.4722
Unclassified_Archaeorhizomycetes	0.3111	0.9078	2.9180	0.3427	0	0	ND	ND
Dothideales	0.3282	0.317	0.9659	1.0353	0.3261	0.135	0.4140	2.4156
Leotiomycetes_oIs	0.3831	0.3278	0.8557	1.1687	1.6885	1.9888	1.1779	0.8490
Hypocreales	0.4379	0.1951	0.4455	2.2445	0.613	0.5174	0.8440	1.1848
Sebacinales	0.5914	1.0266	1.7359	0.5761	5.082	6.3771	1.2548	0.7969
Thelephorales	0.8174	1.1995	1.4675	0.6815	0.8194	1.2092	1.4757	0.6776
Leotiomycetes_unclassified	0.8613	0.4751	0.5516	1.8129	2.5149	2.7485	1.0929	0.9150
Sordariales	1.0054	0.7291	0.7252	1.3790	1.6313	1.1316	0.6937	1.4416
Coniochaetales	1.0488	1.0917	1.0409	0.9607	0.3875	0.2407	0.6212	1.6099
Dothideomycetes_unclassified	1.0549	0.5233	0.4961	2.0159	1.1276	0.5209	0.4620	2.1647
Capnodiales	1.1828	0.3956	0.3345	2.9899	1.5721	0.6083	0.3869	2.5844
Hysteriales	1.2329	2.2568	1.8305	0.5463	1.058	1.212	1.1456	0.8729
Basidiomycota_unclassified	1.3353	1.5253	1.1423	0.8754	0.5237	0.2015	0.3848	2.5990
Atheliales	1.343	2.5103	1.8692	0.5350	1.7828	3.3734	1.8922	0.5285
Sordariomycetes_unclassified	2.0352	2.6896	1.3215	0.7567	0.9267	0.6157	0.6644	1.5051
Pleosporales	2.0434	0.7338	0.3591	2.7847	1.9213	0.9357	0.4870	2.0533
Fungi_unclassified	2.7709	1.9036	0.6870	1.4556	2.5954	0.8036	0.3096	3.2297
Helotiales	3.4413	1.7798	0.5172	1.9335	3.6053	1.9657	0.5452	1.8341
Russulales	3.6784	10.1297	2.7538	0.3631	4.179	12.2744	2.9372	0.3405
Eurotiomycetes_unclassified	3.8466	3.3757	0.8776	1.1395	5.5492	3.4124	0.6149	1.6262
Dothideomycetes_oIs	6.0357	3.492	0.5786	1.7284	7.9399	3.8761	0.4882	2.0484
Eurotiales	7.9922	4.2833	0.5359	1.8659	7.3005	2.9734	0.4073	2.4553
Agaricales	10.3884	8.9115	0.8578	1.1657	8.3053	7.7723	0.9358	1.0686
Ascomycota_unclassified	10.7329	5.3345	0.4970	2.0120	11.3526	3.8325	0.3376	2.9622
Chaetothyriales	15.0499	10.7645	0.7153	1.3981	17.1535	4.5166	0.2633	3.7979
Agaricomycetes_unclassified	17.4891	20.5451	1.1747	0.8513	6.2796	8.626	1.3737	0.7280

Supplementary Table 4. Fungal rRNA ITS Miseq sequencing parameters and statistical estimators in different sampling sites of the gold anomaly and the reference area

Sample	No. of sequences	No. of OTUs	Coverage (%)	Inverse Simpson's	Berger-Parker
Gold anomaly					
NBD01	80603	1762	98.35	10.82	0.21
NBD02	80603	2403	97.88	20.88	0.16
NBD03	80603	1739	98.37	5.49	0.32
NBD04	80603	1149	98.80	1.59	0.79
NBD05	80603	1935	98.28	4.64	0.45
NBD06	80603	2220	97.91	6.88	0.33
NBD07	80603	1690	98.32	4.29	0.47
NBD08	80603	2726	97.43	17.46	0.16
NBD09	80603	1737	98.31	2.18	0.67
NBD10	80603	1331	98.63	1.70	0.76
Reference area					
NBD11	80603	1718	98.35	2.63	0.61
NBD12	80603	1539	98.83	8.63	0.29
NBD13	80603	1796	98.38	9.47	0.26
NBD15	80603	1362	98.88	10.17	0.19
NBD16	80603	1164	98.95	3.11	0.47
NBD17	80603	1802	98.37	5.73	0.33
NBD18	80603	1630	98.48	5.87	0.33
NBD19	80603	1957	98.26	5.82	0.38
NBD20	80603	1359	98.97	3.36	0.53

Supplementary Table 5. Bacterial 16S rRNA gene Miseq sequencing parameters and statistical estimators in different sampling sites of the gold anomaly and the reference area

Sample	No. of sequences	No. of OTUs	Chao	Inverse Simpson's	Shannon
Gold anomaly					
NBD01	1164	767	4110.46	257.64	6.25
NBD02	1164	644	2714.32	153.96	5.92
NBD03	1164	731	3525.40	109.40	6.04
NBD04	1164	765	5583.02	158.22	6.17
NBD05	1164	658	2567.39	75.91	5.75
NBD07	1164	393	1193.28	25.21	4.66
NBD08	1164	606	2444.13	71.29	5.69
NBD09	1164	608	2426.01	80.46	5.72
NBD10	1164	565	2125.71	63.93	5.57
Reference area					
NBD11	1164	648	2812.98	135.28	5.90
NBD12	1164	580	2089.44	110.84	5.71
NBD13	1164	600	2385.03	126.84	5.78
NBD16	1164	534	1839.20	71.96	5.46
NBD17	1164	509	1937.34	81.99	5.42
NBD18	1164	556	2147.34	51.16	5.49

Supplementary Table 6. The difference of bacterial communities' diversity between the gold anomaly and the reference area

	**Gold anomaly vs. Reference**		**Hotspots vs. Reference**	
	Chao	Inverse Simpson's	Chao	Inverse Simpson's
P	0.1821	0.6496	0.1275	0.6403
t, df	t=1.41, df=13	t=0.12, df=13	t=1.73, df=7	t=0.49, df=7
Significant[a]	-[b]	-	-	-

a, P<0.1 was considered statistically significant.
b, + and - represented significant and non-significant, respectively.

Supplementary Table 7. The distribution of the ten most abundant and frequently detected fungal OTUs (determined by 1/CV) in the MENs of the gold anomaly (module a-c) and the reference area (module d-f)

Gold anomaly			Reference area		
Module a	Module b	Module c	Module d	Module e	Module f
Capnodiales	Sordariales		Dothideales	Dothideomycetes_unclassified	Capnodiales
Hypocreales	Trechisporales		Coniochaetales	Sebacinales	Hypocreales
Dothideomycetes_unclassified	Coniochaetales		Sordariales	Thelephorales	
Leotiomycetes_unclassified			Leotiomycetes_unclassified		
Leotiomycetes_oIs*			Leotiomycetes_oIs		
Dothideales					
Thelephorales					

*oIs means order_Incertae_sedis

Supplementary Table 8. Au(III) concentrations for preparing the standard curve for colorimetric measurement

**Target concentration of Au(III) (μM)**	**Real concentration of Au(III) (μM)**	**Absorbance (654 nm)**
2	2.715	0.039
3	4.102	0.081
10	13.794	0.481
20	27.651	1.021
30	42.592	1.583

Supplementary Table 9. Thermodynamic properties of substances[1,2]

	$Au_{(s)}$	$O_2^-{}_{(g)}$	$H^+_{(aq)}$	Au^{3+}	$H_2O_{(l)}$
$\Delta_f H^\Theta$ (kJ·mol^{-1})	0	-43.22	0	405.56	-285.8
S^Θ(J·mol^{-1}·K^{-1})	47.7	209.59	0	-242.25	69.9

Supplementary References

1. Linstrom, P. J. & Mallard, W. G. The NIST Chemistry WebBook: A Chemical Data Resource on the Internet. *J. Chem. Eng. Data* **46**, 1059–1063 (2001).
2. Shock, E. L., Sassani, D. C., Willis, M. & Sverjensky, D. A. Inorganic species in geologic fluids: Correlations among standard molal thermodynamic properties of aqueous ions and hydroxide complexes. *Geochim. Cosmochim. Acta* **61**, 907–950 (1997).
