# Supplementary material for: Evidence for fungi and gold redox interaction under Earth surface conditions
Source: Nat Commun. 2019 May 23;10:2290. doi: 10.1038/s41467-019-10006-5 (PMC6533363; doi:10.1038/s41467-019-10006-5)
Supplement: Supplementary file 3 — Description of Additional Supplementary Files [file 41467_2019_10006_MOESM3_ESM.pdf]

Description of Additional Supplementary Information

File Name: Supplementary Dataset 1

Description: Element composition and concentrations of the samples from the gold anomaly (NBD01-10) and the adjacent area (NBD11-13, NBD15-20). STD.GLG3012-2 is quality control.

File Name: Supplementary Dataset 2

Description: List of the assigned taxonomy name of each fungal OTU.

File Name: Supplementary Dataset 3

Description: List of the network nodes' centrality indexes of the fungal community in the gold anomalous soils.

File Name: Supplementary Dataset 4

Description: List of the network nodes' centrality indexes of the fungal community in the adjacent reference area.
